# Meningococcal Carriage among Adolescents after Mass Meningococcal C Conjugate Vaccination Campaigns in Salvador, Brazil

**DOI:** 10.1371/journal.pone.0166475

**Published:** 2016-11-18

**Authors:** Amélia Maria Pithon Borges Nunes, Guilherme Sousa Ribeiro, Ítalo Eustáquio Ferreira, Ana Rafaela Silva Simões Moura, Ridalva Dias Martins Felzemburgh, Ana Paula Silva de Lemos, Mitermayer Galvão Reis, José Cassio de Moraes, Leila Carvalho Campos

**Affiliations:** 1 Instituto Gonçalo Moniz, FIOCRUZ-BA, 40296–710, Salvador, Brazil; 2 Instituto de Saúde Coletiva, Universidade Federal da Bahia, 40110–040, Salvador, Brazil; 3 Escola de Enfermagem, Universidade Federal da Bahia, 40110–060, Salvador, Brazil; 4 Centro de Bacteriologia, Instituto Adolfo Lutz, 01246–000, São Paulo, Brazil; 5 Faculdade de Ciências Médicas da Santa Casa de São Paulo, 01221–020, São Paulo, Brazil; Public Health England, UNITED KINGDOM

## Abstract

*Neisseria meningitidis* is a commensal bacterium of the human nasopharynx. In rare cases, it penetrates the mucosa, entering the blood stream and causing various forms of disease. Meningococcal conjugate vaccines can prevent invasive disease not only by direct effect in vaccinated individuals but also by herd protection, preventing acquisition of carriage, which interrupts transmission and leads to protection of unvaccinated persons. In 2010 in Salvador, Brazil, an outbreak of group C meningococcal disease led to a mass meningococcal serogroup C conjugate vaccination drive, targeting those <5 and 10–24 years of age. The present study aimed to estimate the prevalence of and identify factors associated with *N*. *meningitidis* carriage among adolescents from Salvador, Brazil, in the post-vaccination period. In spring 2014, we performed a cross-sectional study involving 1,200 public school students aged 11–19 years old. Oropharyngeal swabs were collected to identify *N*. *meningitidis*. Of the 59 colonized participants, 36 (61.0%) carried non-groupable *N*. *meningitidis*, while genogroup B (11.9%), Y (8.5%), E (6.8%), Z (5.1%), C (3.4%), and W (3.4%) were also detected. The overall prevalence of *N*. *meningitidis* carriage was 4.9% (95% confidence interval [CI], 3.6–6.1%); the prevalence of *N*. *meningitidis* genogroup C was 0.17% (95% CI, 0.0–0.40%). There was no difference by age. Factors associated with carriage were having only one, shared, bedroom in the household (PR, 2.02; 95% CI, 0.99–4.12, *p* = 0.05); the mother being the only smoker in the home (PR, 2.48; 95% CI, 1.16–5.29; *p* = 0.01); and going to pubs/parties more than 5 times/month (PR, 2.61; 95% CI, 1.38–4.92; *p* = 0.02). Our findings show that the *N*. *meningitidis* carriage rate in adolescents from Salvador, Bahia, is low and is potentially influenced by the low prevalence of *N*. *meningitidis* genogroup C. However, continued surveillance is important to identify changes in the dynamics of *N*. *meningitidis*, including the emergence of diseases due to a non-C serogroup.

## Introduction

*Neisseria meningitidis* is commonly carried as part of the commensal microbiota in the upper respiratory tract of humans. However, occasionally the bacteria invade the bloodstream and causes severe diseases, such as meningitis and sepsis, which can be fatal or produce permanent neurological sequelae in survivors [1]. Each year, 0.5–1.2 million people are affected by meningococcal disease (MD), and 50,000 to 135,000 die [2]. Most cases are caused by 6 of the 12 recognized capsular groups (A, B, C, W, Y and X), which are defined based on the different immunochemical variants of the polysaccharide capsules produced by the bacteria [3].

The incidence of MD is cyclical in nature and varies geographically and over time [4]. For example, in Latin America, the incidence of MD varies from <0.1 to 2.0 cases per 100,000 inhabitants [5], while in Brazil, the annual incidence of MD before 2010 was stable at 1.5–2.0 cases per 100,000 inhabitants [6].

In addition, between 2007 and 2009, 194 cases of MD, resulting in a case fatality rate of 48%, were identified in the state of Bahia, Brazil. These outbreaks were caused by serogroup C meningococci [7]. In response, the state government initiated mass meningococcal serogroup C conjugate (MCC) vaccination campaigns in 2010 targeting the age groups most affected (<5 and 10–24 years of age). The campaigns proved to be highly effective in controlling the outbreak [8] and more than 611,673 doses of the MCC vaccine were administered, reaching an estimated 92% of children <5 years old, 80.4% of 10–14 year olds, 67.4% of 15–19 year olds, and 41% of 20–24 year olds [9]. Later that year, the Brazilian Ministry of Health introduced the MCC vaccine for children <2 years old as part of the National Immunization Program. The MCC vaccine schedule included two doses, one at 3 months, one at 5 months, and one booster at 12–15 months of age [10].

Although *N*. *meningitidis* has the potential to cause invasive disease, the bacteria usually colonize the human nasopharynx asymptomatically [1] and are transmitted from person to person by aerosol droplets [11]. Colonization can last a few days to several months and can include 3–25% of the population; the highest rates are among adolescents [12]. Several factors may facilitate meningococcal colonization, including active/passive smoking, intimate kissing, crowding, and social deprivation [13]. In contrast, meningococcal conjugate vaccines may reduce acquisition of the vaccine serogroups of *N*. *meningitidis* and therefore reduce transmission of the organism, leading to herd protection of unvaccinated people [14].

Currently, few studies have investigated the prevalence of and factors associated with *N*. *meningitidis* carriage among adolescents in Latin America. Assessing meningococcal carriage in the pre/post-meningococcal vaccine period is critical to understanding the epidemiology and transmission dynamics of *N*. *meningitidis* as well as for assessing the extent and potential for vaccination strategies to induce herd protection [5]. In Salvador, the abrupt decision of State Health Secretary to introduce the MCC vaccine due to an outbreak of MD in the city hampered the conduction of a baseline carriage survey. However, it provided a unique opportunity to study the frequency of *N*. *meningitidis* carriage among the sole population of adolescents exposed to a MCC mass vaccination campaign in Brazil. Therefore, the aims of this study were to estimate the prevalence of and identify factors associated with *N*. *meningitidis* carriage among adolescents in Salvador, Brazil, in the post-vaccine period.

## Methods

### Ethical considerations

This study was approved by the Ethics Committee of the Instituto Gonçalo Moniz, FIOCRUZ-BA (CAAE #16099713.1.0000.0040). Written informed consent from all participants (or guardians) in the study were obtained before sample and data collection.

### Study design and participant selection

A cross-sectional study was conducted between September and December 2014 in the city of Salvador (estimated population 2.9 million in 2014), the capital of the state of Bahia. Study participants were selected from students aged 11–19 years enrolled in municipal or state public schools during the current school year. Participants were chosen by a probabilistic, two-stage selection process. In the first stage, we randomly selected 155 schools from the 660 municipal and state schools operating in Salvador in 2014, weighting the probability of school selection by the proportion of students aged 11–19 years in each of the city's schools. Of the 155 selected schools, 134 provided lists with the names of the students per class and were, therefore, included in the second stage of the selection process. In this stage, we used a computer-based program to generate random numbers from each class list, limiting the maximum number of selected students per class to five to avoid clustering of participants in the same classroom. The required sample size (*n* = 1,200) was based on a hypothetical prevalence of *N*. *meningitidis* carriage of 9.9% (as obtained in a study with adolescents in schools in Campinas, Brazil [15], a precision of 2% standard deviation for a 95% confidence interval, and a correction factor for the cluster design effect of 2. However, because we expected that about half of the selected participants would refuse to participate in the study, we invited 2,440 students to participate.

### Data collection

A trained research team interviewed the participants using a standardized questionnaire to collect data on age, sex, self-reported race/skin colour, place of residence, and grade. Information on the following potential risk factors was also obtained: number of household residents, number of rooms used for sleeping in the home, mother’s level of education, passive/active smoking, influenza-like symptoms in the past 15 days, use of antibiotics in the past 15 days, going to pubs/parties in the past month, and having ever received the MCC vaccine. Study data were collected and managed using the REDCap (Research Electronic Data Capture) electronic data capture tool [16] hosted at Instituto Gonçalo Moniz.

### Sample collection and bacterial identification

The posterior pharyngeal wall behind the uvula of each volunteer was swabbed using a sterile rayon swab. The swab was immediately plated onto a selective agar medium (modified Thayer-Martin vancomycin, colistin, nystatin, and trimethoprim) and introduced in plastic tubes containing 1 mL of skim milk-tryptone-glucose-glycerine (STGG) transport medium [17]. The samples were sent to the Instituto Gonçalo Moniz within 4 h after collection. In the laboratory, STGG tubes were stored at -80°C until further analysis. Thayer-Martin plates were incubated at 37°C with 5% CO_2_. After 24–48 hours of incubation, the plates were inspected, and colonies with characteristics of *Neisseria sp*. were sub-cultured on blood agar medium for species identification by Gram staining, oxidase reaction, and carbohydrate utilization tests. Results were confirmed by API-NH® strips (bioMérieux, Hazelwood, MO, USA). Samples positive for *N*. *meningitidis* were genogrouped by polymerase chain reaction with specific primers for detecting genogroups A, B, C, W, and Y [18]. Identification of genogroups E and Z was performed using whole-genome sequencing [19] conducted at the Meningitis Laboratory, Centers for Disease Control and Prevention, Atlanta, USA.

### Statistical analysis

Data were cleaned, validated, and analysed using STATA 12 statistical software (College Station, TX, USA). The prevalence of *N*. *meningitidis* carriage was calculated for the total sample and for subgroups (sex, age, race/skin colour, grade, and potential risk factors). Quantitative variables were summarized using means and standard deviations or medians and interquartile ranges, as appropriate. Categorical variables were described using percentages. To identify exposures associated with *N*. *meningitidis* carriage, bivariate analyses were performed, and prevalence ratios (PR) with the respective 95% confidence intervals (95% CI) were reported. The significance level was set at *p* ≤ 0.05.

## Results

### Study participants

A total of 1,200 participants were included in the study. Of them, 415 (34.6%) were 11–13 years old, 382 (31.8%) were 14–16 years old, and 403 (33.6%) were 17–19 years old. Most of the participants were female (61.8%). The majority (60.0%) had 6–9 years of education, and 93.5% of the participants reported their mothers’ level of education to be less than a college degree. The median number of people sleeping in the same room was 1.7 (interquartile range, 1.5–2.0). Of the 732 participants who provided information on MCC vaccination, 162 (22.1%) responded that they had been vaccinated (Table 1).

**Table 1:** Socio-demographic characteristics of students enrolled in the *Neisseria meningitidis* pharyngeal carriage study in Salvador, Brasil, in 2014.

Characteristic	n (%) or median (IQR)
***Female sex***	741 (61.8)
***Age group:***	
11–13 years	415 (34.6)
14–16 years	382 (31.8)
17–19 years	403 (33.6)
***Self-reported skin colour:***	
White	88 (7.3)
Black	424 (35.3)
Mixed	610 (50.8)
Other	78 (6.5)
***Grade:***	
1–5	74 (6.2)
6–9	720 (60.0)
10–12	406 (33.8)
***Mother’s education level:*** ^*^^†^	
Less than high school	353 (39.2)
High school	489 (54.3)
More than high school	59 (6.6)
***No*. *of residents per room***^*†*^	1.7 (1.5–2.0)
***Reported MCC vaccination***^*†*^	162 (22.13)

* For participants whose mothers were not the legal guardian, data on education level was collected for the primary caregiver.

† Data on mother’s education level, number of residents per room, and MCC vaccination were available for 901, 1,194, and 732 participants, respectively.

Abbreviations: No, number; IQR, interquartile range; MCC, meningococcal C conjugate vaccination.

### Prevalence of and factors associated with *N*. *meningitidis* carriage

Overall, 59 of the 1,200 participants were colonized by *N*. *meningitidis*, representing a carriage prevalence of 4.9% (95% CI, 3.6–6.1%). There was no difference in the prevalence by age group. The prevalence among participants who reported only having one room for sleeping was 2.02 times greater (95% CI, 0.99–4.12, *p* = 0.05) than those who reported having two or more rooms for sleeping. None of the colonized participants reported being active smokers, but several had passive exposure to smoke in the home. Compared to participants who were not exposed to cigarette smoke in their homes, those who reported household exposure to smoking had a 1.40 (95% CI, 0.80–2.43; *p* = 0.23) times greater prevalence of *N*. *meningitidis* carriage. Those participants who reported that only their mothers or siblings smoked had a 2.48 (95% CI, 1.16–5.29; *p* = 0.01) and 2.31 (95% CI, 0.60–8.88; *p* = 0.22) times greater prevalence, respectively. Those who had gone to a pub/party had a 1.61 (95% CI, 0.96–2.67; *p* = 0.06) times greater prevalence of *N*. *meningitidis* carriage compared to those who had not. In addition, a gradient for the prevalence of colonization was observed according to the frequency of going to a pub/party. Those who went 1–4 times per month had a higher prevalence (PR, 1.28; 95% CI, 0.72–2.28; *p* = 0.38) than those who did not go at all, while those who went ≥5 times per month had an even higher prevalence rate (PR, 2.61; 95% CI, 1.38–4.92; *p* = 0.02). Moreover, *N*. *meningitidis* carriage was lower for participants whose mothers had at least a high school education (PR, 0.58; 95% CI, 0.32–1.05, *p* = 0.07) than those whose mothers had less than a high school education (Table 2).

**Table 2:** Prevalence of and factors associated with *Neisseria meningitidis* pharyngeal carriage among students in Salvador, Brazil, in 2014.

Characteristics	No. of participants	*N*. *meningitides* carriage, n (%)	Prevalence ratio (95% CI)	*p* value
***Age group:***				
11–13 years	415	19 (4.57)	1.00	
14–16 years	382	20 (5.23)	1.14 (0.62–2.10)	0.67
17–19 years	403	20 (4.96)	1.08 (0.59–2.00)	0.79
***Sex:***				
Female	741	40 (5.39)	1.00	
Male	459	19 (4.13)	0.76 (0.45–1.30)	0.32
***Self-reported skin colour:***				
White	88	6 (6.81)	1.00	
Black	424	14 (3.30)	0.48 (0.19–1.22)	0.12
Mixed	610	33 (5.40)	0.79 (0.34–1.83)	0.59
Other	78	6 (7.69)	1.12 (0.37–3.35)	0.82
***Grade:***				
1–5	74	3 (4.05)	1.00	
6–9	720	37 (5.13)	1.26 (0.40–4.01)	0.68
10–12	406	19 (4.68)	1.15 (0.35–3.80)	0.81
***Mother’s education level:*** ^*^				
Less than high school	353	22 (6.23)	1.00	
High school or more	548	20 (3.65)	0.58 (0.32–1.05)	0.07
***No*. *of residents:***				
1–3	423	21 (4.96)	1.00	
4–6	677	34 (5.02)	1.01 (0.59–1.72)	0.96
≥7	99	4 (4.04)	0.81 (0.28–2.31)	0.69
***No*. *of residents per room:***				
<1.9	601	24 (4.00)	1.00	
2–2.9	464	27 (5.81)	1.45 (0.85–2.49)	0.16
≥3	135	8 (5.92)	1.48 (0.68–3.23)	0.31
***No*. *of household rooms used for sleeping:***				
Only one	86	8 (9.30)	2.02 (0.99–4.12)	0.05
Two or more	1108	51 (4.94)	1.00	
***Reported prior use of MCC vaccine:***				
Yes	162	6 (3.70)	1.00	
No	570	35 (6.14)	1.65 (0.70–3.87)	0.23
***Household exposure to cigarette smoke:***				
Yes	251	16 (6.37)	1.40 (0.80–2.45)	0.23
No	947	43 (4.54)	1.00	
***Who smokes in the household?***				
No smokers	947	43 (4.54)	1.00	
Only father	60	2 (3.33)	0.73 (0.18–2.96)	0.66
Only mother	62	7 (11.29)	2.48 (1.16–5.29)	0.01
Only sibling	19	2 (10.52)	2.31(0.60–8.88)	0.22
>1 smoker	32	2 (6.25)	1.37 (0.35–5.43)	0.64
***Going to pubs/parties:***				
Yes	569	35 (6.15)	1.61 (0.97–2.67)	0.06
No	628	24 (3.82)	1.00	
***Pubs/parties per month:***				
<1	628	24 (3.83)	1.00	
1–4 times	427	21 (4.91)	1.28 (0.72–2.28)	0.38
≥5 times	140	14 (10.00)	2.61 (1.38–4.92)	0.02
***Influenza-like illness in the past 2 weeks:***				
No	682	37 (5.42)	1.00	
Yes	517	22 (4.25)	0.78 (0.46–1.31)	0.35
***Antibiotic use in the past 2 weeks:***				
No	1140	57 (5.00)	1.00	
Yes	55	2 (3.63)	0.72 (0.18–2.90)	0.64
***N*. *lactamica carriage:***				
No	1146	58 (5.06)	2.73 (0.39–19.4)	0.48
Yes	54	1 (1.85)	1.00	

^*^ For participants whose mothers were not the legal guardian, data on education level was collected for the primary caregiver.

Abbreviations: No, number; MCC, meningococcal C conjugate vaccination; CI, confidence interval.

### *N*. *meningitidis* genogroups

Of the 59 colonized participants, 36 (61.0%) carried non-groupable *N*. *meningitidis*, while 7 (11.8%) carried genogroup B, 5 (8.5%) genogroup Y, 4 (6.7%) genogroup E, 3 (5.1%) genogroup Z, 2 (3.4%) genogroup C, and 2 (3.4%) genogroup W. None of the two participants colonized by genogroup C reported prior use of the MCC vaccine. The prevalence of colonization by *N*. *meningitidis* genogroup C was 0.17% (95% CI, 0.00–0.40) of the participants. We also identified the isolation of *N*. *lactamica* in 54 (4.5%) of 1,200 subjects. One participant carried both *N*. *meningitidis* and *N*. *lactamica*. Participants who were not colonized by *N*. *lactamica* had an increased prevalence of *N*. *meningitidis*, but this difference was not statistically significant (PR, 2.73; 95% CI, 0.39–19.4; *p* = 0.48) (Table 2).

## Discussion

The results of this study showed that the overall prevalence of *N*. *meningitidis* carriage after the mass MCC vaccination campaign was 4.9%, while carriage of *N*. *meningitidis* group C was 0.17%. The overall prevalence for adolescents in Salvador was lower than the prevalence (9.9%) observed in a similar study performed in Campinas, Brazil, after introducing the MCC vaccine among children <2 years old [15]. Moreover, our results were similar to those of studies from other Latin American countries, such as Colombia (6.85%) [20] and Chile (6.5%) [21].

The relatively low prevalence of *N*. *meningitidis* carriage found in this study may be due to the mass vaccination campaigns against *N*. *meningitidis* serogroup C conducted in Salvador, in particular because the vaccination campaigns also targeted adolescents and young adults [7]. The finding that only 3.4% of the *N*. *meningitidis* isolates were from genogroup C and that the prevalence of this genogroup among the participants was 0.17% help to support this hypothesis. Moreover, in a similar study conducted in Campinas, Brazil, the prevalence of genogroup C among adolescents was 1.32%; almost eight times higher than the prevalence rate of MenC carriage found in our study. This difference also suggests that the vaccination of the 10–25-year-old population group during the meningococcal C outbreak in Salvador might have influenced the genogroup C carriage rate as an effect of vaccination.

Furthermore, the immunization program may have also helped to prevent transmission and colonization of the organism in non-vaccinated individuals through herd protection [22]. This is supported by a case-control study showing that mass MCC vaccination of target groups proved effective in preventing serogroup C MD in Salvador, Brazil [9], as well as by a study in the UK (the first country to introduce MCC vaccination) showing that carriage of serogroup C meningococci was reduced by 66% among students aged 15–17 years one year after introduction of the vaccine [8].

On the other hand, even though meningococcal carriage is an age-dependent phenomenon (increasing from 4.5% in infants to 23.7% in 19 year olds and then decreasing to 7.8% in adults over 50 years old [23]), we did not find any age-related difference in the carriage prevalence among adolescents 11–19 years old, results that are similar to those found in Campinas [15]. In addition, even though men are the most prevalent meningococcal carriers worldwide [13], sex was not associated with meningococcal carriage in our study [15, 20].

In this study, most of the colonized subjects carried non-groupable *N*. *meningitidis* (61%) followed by genogroup B (11.8%). These findings are in accordance with other studies showing that most *N*. *meningitidis* isolates in carriers are non-groupable [15, 24]. Although non-groupable *N*. *meningitidis* strains are not generally associated with invasive disease, in rare instances capsule-deficient strains have been isolated from subjects with MD [25]. Furthermore, *N*. *meningitidis* is genetically competent, so even seemingly harmless capsule-deficient strains can recombine to become virulent [26].

It is important to note that most of the capsular groups related to invasive diseases (B, C, W, and Y) [27] were found among the population studied. Although the carriage rate of genogroup W was low (0.17%), these strains were characterized as belonging to the hypervirulent sequence type ST-11 genetic lineage, and as having the PorA antigen-encoding gene type P1.5,2 [28, 29]. Recently, we have noticed a rapid spread of MenW endemic hypervirulent sequence type (ST) 11 clonal complex in England and Wales, South America, and South Africa [29]. It is important to highlight that the number of *N*. *meningitidis* serogroup W ST-11 cases has increased in Brazil [5, 6, 30]. Moreover, the fact that genogroup B was the most frequent groupable isolate found highlights the necessity for continuous MD surveillance to detect any changes in the incidence of meningococcal serogroup B invasive diseases in the future. In fact, serogroup B has been the second leading cause of disease in Brazil since 2007 [31, 32] and, until 2011, MenB was the leading cause of meningococcal disease in Chile [21, 33].

The bivariate analysis in this study showed that number of household rooms used for sleeping, passive smoking, and going to pubs/parties were associated with meningococcal carriage among adolescents [15, 34]. As observed in previous studies, sharing rooms to sleep may increase meningococcal carriage because individuals tend to be closer to one another than usual, facilitating the transmission of *N*. *meningitidis* [35, 36]. Going to pubs/parties has also been associated with *N*. *meningitidis* carriage [27], most likely because loud music and overcrowding at pubs/parties drives people to get closer and speak louder, increasing the likelihood of bacterial transmission [37].

On the other hand, although other studies have found an association between meningococcal carriage and active smoking [38, 39], we did not. However, the number of smokers in our study was very small, possibly limiting the power to detect an association. Nonetheless, a positive association was found among carriers who reported that only their siblings or only their mother smoked in the household, the latter being statistically significant. This may be because cigarette smoke causes damage to the ciliary activity in the nasopharynx, increasing susceptibility to bacterial infection and, thus, facilitating colonization by *N*. *meningitidis* [39]. Furthermore, because mothers may spend more time with their children during childhood and adolescence, maternal smoking might increase the probability of meningococcal colonization [37].

Moreover, although not statistically significant, carriage was 42% lower for participants whose mothers had at least a high school education when compared to those whose mothers had less than a high school education, an association that has also been observed in other studies [13, 15]. This may be because parental level of education plays an important role in determining participant socio-economic condition, a factor associated with meningococcal carriage in other studies [15, 27].

We also found that adolescents colonized by *N*. *lactamica* had a lower prevalence of *N*. *meningitidis* carriage than those not colonized by *N*. *lactamica*, but the difference was not statistically significant. This is most likely because *N*. *lactamica* works as a protective agent against meningococcal colonization either by stimulating immune response from the host or by competing for the same ecological niche [40].

Although this study has several strengths, there are also some limitations. First, no meningococcal carriage studies were conducted before the mass vaccination campaigns; therefore, we could not measure the impact of the MCC vaccine on carriage over time. Second, only students from public schools in Salvador were included while students in private schools were excluded. As students from public schools usually have a lower socioeconomic status, which is associated with meningococcal carriage, the prevalence of *N*. *meningitidis* colonization may have been overestimated in this study. Furthermore, most of the participants did not provide documentation confirming whether they had received the MCC vaccine, hampering the analysis regarding the influence of prior vaccination on *N*. *meningitidis* carriage. Finally, as the prevalence of *N*. *meningitidis* was low, our analyses did not have enough power to detect more statistically significant associations.

Nonetheless, the evidence gathered during this study indicates that adolescents living in a Brazilian city where mass MCC vaccination was instituted had a low prevalence of *N*. *meningitidis* colonization, particularly for serogroup C. Maintaining low MenC carriage rates is critical to control MenC re-emergence. Therefore, continued surveillance is needed to detect possible changes in the dynamics of *N*. *meningitidis* colonization and/or the emergence of a given capsular group, to aid defining the need of a booster dose of MCC vaccine, and to inform about the potential benefit of further introduction of Men ACWY conjugate or MenB protein vaccines.
